# Thinking out of the box: revisiting health surveillance based on medical records

**DOI:** 10.1017/ash.2023.451

**Published:** 2023-10-24

**Authors:** Vanderson S. Sampaio, Rafael Lopes, Mina Cintho Ozahata, Helder I. Nakaya, Erick Sousa, José D. Araújo, Marcelo A.S. Bragatte, Anderson F. Brito, Regina Maura Zettoni Grespan, Maria Ligia Damato Capuani, Helves Humberto Domingues, Alessandra Cristina Guedes Pellini, Sheila de Oliveira Garcia Mateos, Mônica Tilli Reis Pessoa Conde, Fabio Eudes Leal, Ester Sabino, Mariangela Simão, Jorge Kalil

**Affiliations:** 1 Instituto Todos pela Saúde, São Paulo, Brazil; 2 Hospital Israelita Albert Einstein, São Paulo, Brazil; 3 Fundação de Medicina Tropical Dr. Heitor Vieira Dourado, Manaus, Brazil; 4 School of Health Sciences, Amazonas State University, Manaus, Brazil; 5 Laboratory of Clinical Immunology and Allergy-LIM60/University of Sao Paulo School of Medicine, São Paulo, Brazil; 6 Institute for Investigation in Immunology - iii-INCT, São Paulo, Brazil; 7 Laboratory of Immunology, Heart Institute, University of São Paulo School of Medicine, São Paulo, Brazil; 8 Instituto de Medicina Tropical da Faculdade de Medicina da Universidade de São Paulo, São Paulo, Brazil; 9 Municipal University of São Caetano do Sul, São Caetano do Sul, São Paulo, Brazil; 10 Telehealth Group, School of Medicine, Federal University of Goiás, Goiás, Brazil; 11 Capixaba Institute for Teaching, Research and Innovation in Health (ICEPi), Espírito Santo, Brazil; 12 Secretary of Health of The Municipality of São Caetano do Sul, São Paulo, Brazil; 13 Modular Research System Ltda (MRV), Brazil; 14 Nove de Julho University (UNINOVE), São Paulo, Brazil

## Abstract

Despite the considerable advances in the last years, the health information systems for health surveillance still need to overcome some critical issues so that epidemic detection can be performed in real time. For instance, despite the efforts of the Brazilian Ministry of Health (MoH) to make COVID-19 data available during the pandemic, delays due to data entry and data availability posed an additional threat to disease monitoring. Here, we propose a complementary approach by using electronic medical records (EMRs) data collected in real time to generate a system to enable insights from the local health surveillance system personnel. As a proof of concept, we assessed data from São Caetano do Sul City (SCS), São Paulo, Brazil. We used the “fever” term as a sentinel event. Regular expression techniques were applied to detect febrile diseases. Other specific terms such as “malaria,” “dengue,” “Zika,” or any infectious disease were included in the dictionary and mapped to “fever.” Additionally, after “tokenizing,” we assessed the frequencies of most mentioned terms when fever was also mentioned in the patient complaint. The findings allowed us to detect the overlapping outbreaks of both COVID-19 Omicron BA.1 subvariant and Influenza A virus, which were confirmed by our team by analyzing data from private laboratories and another COVID-19 public monitoring system. Timely information generated from EMRs will be a very important tool to the decision-making process as well as research in epidemiology. Quality and security on the data produced is of paramount importance to allow the use by health surveillance systems.

## Background

The Brazilian Unified Health System (SUS) was created in 1990 to implement the 1988 Brazilian Constitution health component, which established universal health access, including all aspects, as a fundamental right.^1^ Although part of a unified system, health information systems (*HIS*) are historically part of specific verticalized control programs like malaria, tuberculosis, and hepatitis, which developed as independent initiatives in the past. Therefore, health information used to be fragmented into distinct databases.^2^

An important landmark happened in 1975, when the Ministry of Health (MoH) promoted the first meeting on HISs to discuss the implementation of the main *HIS*.^3^

Thereafter, Brazilian *HIS* have substantially increased in quality, mainly in the last few years with the migration to web-based services enabling fast and secure data transfer from municipalities to the MoH’s servers and providing access to summary reports. These *HIS* allowed data collection in a patient-level granularity, allowing researchers, health professionals, and policymakers to conduct ecological and individual analyses.^3^

Among those *HIS*, we emphasize the Information System of Diseases Notification (SINAN), which stores data from all mandatory notification diseases, the Information System of Live Births (SINASC), responsible for collecting and storing data from all live births, and the Information System of Mortality (SIM), where death registers are collected and stored. Others *HIS* were developed for specific needs, such as SIVEP malaria (used to collect and store data of malaria infection in the Amazon region) and SIVEP *gripe*, used to collect and store data of severe acute respiratory syndrome in the whole country.

Despite the considerable advances in the last years, those *HIS* still need to overcome some critical issues, like the absence of a primary key to enable data from the same person to be linked quickly from the different databases. More essential, difficulties on broad Internet access poses an additional barrier to the timely availability of data so that epidemic detection can be performed in real time. Additionally, MoH’s open data policies are still unclear, sometimes only a byproduct of specific research projects. For instance, despite the efforts of the MoH to make COVID-19 data available during the pandemic, delays due to both the data entering and data availability are still observed. A chain of events in the notification process, which are also sources of delay, has posed an additional obstacle to monitor the pandemic in real time.^4^

Responses to epidemics require the ability to detect signs and symptoms quickly, even if the pathogen is unknown. The existing *HIS* are not thought to allow prompt epidemic detection because of their nature and limitations.^4^

In recent years, medical records are increasingly moving toward electronic platforms. These clinical registers are a rich source of patient information and epidemiological information, allowing for timely data, in-depth investigation of patient condition, follow-up, and sample availability for further analysis.^5^

**Figure 1.**
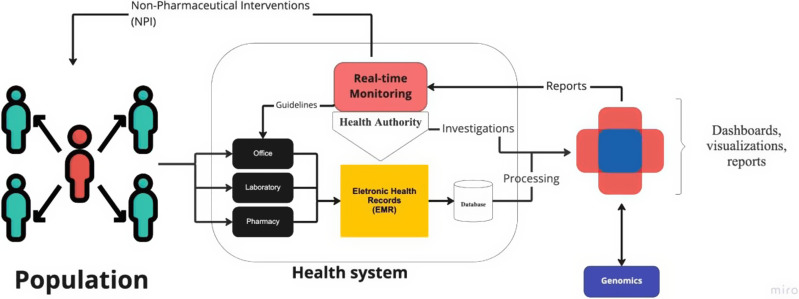
Flowchart of surveillance process based on electronic medical records.

Here, we propose a complementary approach by using EMRs data collected from health units in real time to generate a user-friendly interface with visual analyses to enable insights from the local health surveillance system personnel. Details on the whole process can be viewed in Figure 1.

## Methods

A pilot project was proposed to be implemented in São Caetano do Sul City (SCS), which is part of the metropolitan region of São Paulo with an estimated population of 162,763 inhabitants, and the highest human development index (HDI) in Brazil (0,862). SCS has an integrated health system which includes information for all health facilities levels, from the basic health unit to hospitals. Also, the city presents a higher rate of chronic diseases and eventually outbreaks of infectious diseases.^6,7^

SCS has implemented EMR systems in the main hospitals and medium-level health units. Here, we used data from MV systems, medical records systems from *Secretaria Municipal de Saúde de São Caetano do Sul* (SMS-SCS). MV is a broad-purpose third-party system developed to collect and manage hospital data. This system provides real-time data availability on medical charts, treatment, clinical management, and diagnostic results. Additionally, in April 2022, in the face of an urgent/emergency scenario related to the COVID-19 pandemic, the Corona São Caetano Platform (CSCP) was implemented. The CSCP is an online platform for managing all activities and actions related to COVID-19, aiming promoting assistance to cases of COVID-19, combining remote care and home visits to collect samples for diagnosis, and integrating laboratory and clinical data related to the etiological diagnosis. The data on the suspected cases of COVID-19 are structured in an electronic database stored on servers with restricted and secure access.^6^

As proof of concept, we chose “fever” as a sentinel event. Regular expression techniques were applied to allow the algorithm to recognize any word or expression that states febrile diseases. Other specific terms such as “malaria,” “dengue,” “Zika,” or any infectious disease were included in the dictionary and mapped to “fever.” Additionally, after “tokenizing,” we assessed the frequencies of most mentioned terms when fever was also mentioned in the patient complaint. The tokenization process consists of split words as distinct registers from a text field in a dataset. Additionally, *stopwords* as articles, prepositions, and verbs were excluded from the processed dataset.^8,9^

To reach real-time non-identified data from medical records, APIs were made available by the local team. Through these interfaces, we developed pipelines to collect, process, and provide visual analyses available in a dashboard. Both the processing and visual analyses were carried out by using R software (v. 4.2.1) and RStudio IDE (v. 2022.07.1). Additionally, the following libraries were used: *flexdashboard, dplyr, lubridate, pyramid, ggplot2, bslib, stringr, wordcloud2, sf, tidytable, knitr, prophet, dygraphs, tidytext,* and *stringi*. The scripts, as well as nonsensitive sample data, can be found at a Github project link: https://github.com/InstitutoTodosPelaSaude/SCS.

## Case

As a case description, here we present a dashboard and respective findings from São Caetano do Sul, São Paulo.^10^

Data from a hospital and a medium complexity health unity from SCS were assessed. In 2022, from January to September, almost 120,000 medical appointments were performed in the two health facilities, with a daily average of almost 4,000. Out of the total registers, 11,400 had mention of “fever” in the patient complaint register. Most of the population were women with ages ranging from 20 to 49 years.

**Figure 2.**
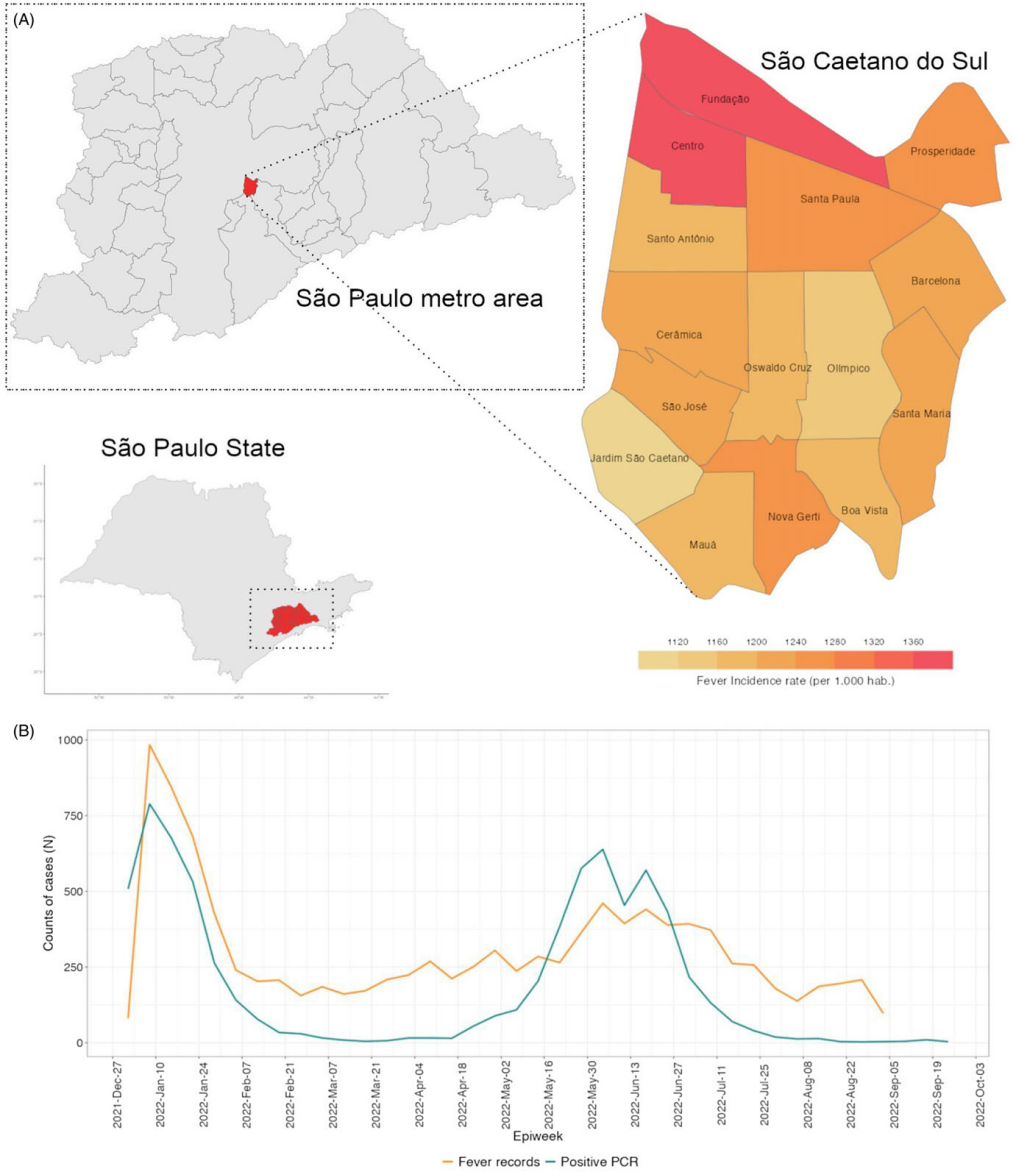
(A) Fever incidence rate per neighborhood (per 1.000 hab.), in São Caetano do Sul. (B) Time series of case counts relating “fever” as a major symptom and positive COVID-19 cases confirmed by RT-PCR in The Corona São Caetano Platform (CSCP).

In the whole period, the most frequent terms mentioned when fever was also detected were pain in the throat, cough, headache, and coryza, with little variation within the last 6 months. The fever rates ranged from 1,050 to 1,025 per 1,000 inhabitants, and the highest were in *Fundação* and *Centro* neighborhoods, in the northern region of the city (Figure 2A). The fever rates, higher than the neighborhood’s population, can be explained by both the quality of the SCS health system and the proximity to São Paulo, the Capital.

Figure 2B shows the time series of fever relating cases, which reveals a bimodal pattern, probably describing the Omicron BA.1 outbreak (weeks 1–6) and the Influenza A outbreak (weeks 20–30) through January–February 2022. The main diagnostic hypotheses in the medical records of the first epidemiological weeks of 2022 pointed out the ICD J111 (Influenza – flu – with other respiratory manifestations, caused by unknown viruses), followed by B342 (infection by unspecified coronavirus). These findings raised flags to the overlapping outbreaks of both COVID-19 Omicron BA.1 subvariant and Influenza A virus, which were confirmed by our team by analyzing data from private laboratories and CSCP.^6,11^

## Discussion

Several studies had been conducted on electronic health systems assessment. Most of them focus on the patient care by itself or propose new systems using different technologies or approaches, which implies additional costs or long learning curves to the public administration, as well as to the health workers. Here, we propose a new approach based on data from health systems, which despite not being designed for that purpose, aggregate a rich amount of data that can be used for decision-making on health surveillance.

The Brazilian MoH currently has to deal with more than 800 unconnected systems from the universal access health system, and most studies have been focusing on proposing new systems.^2^ Besides being a tough challenge to gather these data, and generate useful information, data availability has to be timely to be able to produce actionable information.

The extensive data wrangling capacity needed to process those databases to be ready-to-use exhausted much of the time of the MoH team, further contributing to delays on data availability. This creates a culture of lack of validation of the information generated, posing additional difficulties on the much-needed evidence-based decision-making. In this regard, we strongly suggest the use of data already available through gathering and wrangling the data from the EMRs, from existing systems, without changing the processes or routines that have been functional.

Although not extensively validated, we were able to detect Omicron VOCs and Influ A outbreaks in SCS. The adoption of EMRs has been a trend in the health units due to the high Internet availability.

In the systematic review by Kruse et al., 2018, that wider adoption as well as the increasing interoperability of the electronic system have the potential to strengthen health surveillance and disease prevention.^12^ Here we showed, by using available technology, that it is possible to point out local outbreaks before the official surveillance system.

However, it can be costly and time-consuming to process the amount of data generated by medical records, which were not projected for such epidemiological analysis. Thus, the minimum approach should be less time-consuming. Medical records encompasses a great variety of data on patient follow-up, including clinical and laboratorial reports that can be used in projects such as the one presented here. Also, despite the unstructured data nature, EMRs are less prone to delays than the traditional surveillance structured data. Structuring process introduces more steps on turning the data into information, with less structured data less steps are needed to process it, although giving nonspecific data, here fever relating cases.

The data gathered from medical reports can increase the quality of data, including anamnesis and accurate classification referring to ICD codes or even patients’ complaints. Such an approach can add empowerment to local authorities with prompt information in the process of decision-making, which increases the velocity and accuracy to deal with outbreaks.

Most of the surveillance systems are filled by nonmedical professionals with little or even no expertise in clinical anamnesis. By using medical records, the accuracy of the suggested diagnostics tends to be better than those made by other nonmedical professionals.

Also, the data security of using medical records for epidemiological purposes needs to be considered. Beard et al., 2012, recalls the high demand for data and systems not prepared to meet data security basic requirements. Although progress has been made recently, it is still a main concern in the field when applying algorithms, pipelines of data wrangling and analysis, on data that has met anonymization principles before its usage.^13^

Therefore, preparedness can be built by teams and infrastructure that have the capacity to detect any anomaly referring to outbreaks caused by emerging and re-emerging pathogens. Making data fully available, only, will not be an end-most solution. However, the above strategy can work as an excellent sentinel surveillance system to detect early signs of new outbreaks.

## Final remarks

Synergy between private sector health units and governments must be stimulated to the wide use and implementation of such systems of EMRs. Therefore, companies developing those systems could fill the gap of harmonizing and structuring databases for EMRs. To do so, the government should provide guidelines on those database structures. Timely information generated from EMRs will be a very important tool to the decision-making process as well as research in epidemiology. Quality and security on the data produced is of paramount importance to allow the use by health surveillance systems.
